# Probiotic Potential of Lactic Acid Starter Cultures Isolated from a Traditional Fermented Sorghum-Millet Beverage

**DOI:** 10.1155/2020/7825943

**Published:** 2020-08-04

**Authors:** Stellah Byakika, Ivan Muzira Mukisa, Yusuf Byenkya Byaruhanga, Charles Muyanja

**Affiliations:** Department of Food Technology and Nutrition, School of Food Technology Nutrition and Bioengineering, College of Agricultural and Environmental Sciences, Makerere University, P O Box 7062, Kampala, Uganda

## Abstract

The purpose of this study was to establish the probiotic potential of lactic acid bacteria (LAB) starter cultures, *Lb. plantarum* MNC 21, *L. lactis* MNC 24, and *W. confusa* MNC 20, isolated from a traditionally fermented sorghum-millet beverage from Uganda. The cultures were examined for tolerance to acid and bile salts, bile salt hydrolase (BSH) activity, antibiotic susceptibility, biogenic amine production, mucin degradation, hydrophobicity, auto-aggregation, adherence to the ileum, coaggregation, and antimicrobial properties against selected pathogenic species. *Lb. rhamnosus* yoba 2012, a known probiotic, was the reference. The isolates were tolerant to acid (pH = 3) and bile (1%). *W*. *confusa* MNC 20 and *Lb*. *plantarum* MNC 21 exhibited medium BSH activity (11–15 mm diameter of hydrolysis zone) while *L*. *lactis* and *Lb*. *rhamnosus* yoba 2012 exhibited low BSH activity (<10 mm diameter of hydrolysis zone). All isolates lacked mucolytic activity. *Lb*. *plantarum* MNC 21 and *W*. *confusa* MNC 20 produced agmatine. The candidate and reference microorganisms were resistant to 10 of 21 and 5 of 21 antibiotics, respectively. The isolates exhibited hydrophobic, auto-aggregation and coaggregation properties. These three properties were exhibited more (*p* < 0.05) by the reference than the potential probiotics. The ability of the potential probiotics to attach onto the goat ileum (7.3–8.0 log cfu/cm^2^) was comparable to that of *Lb*. *rhamnosus* yoba 2012 (7.6 log cfu/cm^2^). The four LAB inhibited *E. coli*, *S. aureus*, and *S. enterica* to the same extent (*p* < 0.05). The findings indicated potential probiotic activity of the starter cultures. However, further *in vivo* examination of these isolates is required to confirm their probiotic capabilities.

## 1. Introduction

There is a general global interest in the use of probiotics in food, in feeds, and as supplements to enhance human and animal health. Probiotics are live microorganisms which when administered in adequate amounts confer health benefits to the host [1]. Some of the health benefits include the following: prevention of antibiotic related diarrhea, treatment of irritable bowel syndrome, production of B vitamins, prolongation of life, production of antioxidants and other geroprotectors, serum cholesterol reduction, prevention of cancers, treatment of *Helicobacter pylori*, relief from lactose intolerance, and improved immune response, among others [2–10].

The major bacterial probiotics used in functional foods are lactic acid bacteria (LAB) and *Bifidobacteria* [11]. In fact, majority of probiotic research is based on these two groups, given their association with human health, and generally regarded as safe (GRAS) status [12]. Traditional fermented foods exhibit a rich biodiversity of microorganisms from which probiotic microorganisms can be selected [12, 13]. Indeed, many studies have reported a number of probiotic and potentially probiotic microorganisms from various fermented foods including *kule naoto*, *bryndza* cheese, *hukati*, *hidal, dadhi*, and *dangke* [14–20].

*Obushera* is a traditional fermented sorghum-millet beverage originally from south western Uganda. The beverage is used as a weaning food, thirst quencher, and social drink at gatherings [21]. Traditionally, it is fermented using wild microorganisms, with LAB being among the dominant species involved [22, 23]. LAB are known to contribute to the flavor profile and safety of fermented foods as well as promote health by acting as probiotics [24, 25]. Three LAB isolates from *Obushera*, *Lactobacillus (Lb.) plantarum* MNC 21, *Lactococcus (L.) lactis* MNC 24, and *Weissella(W.) confusa* MNC 20, possess excellent starter culture properties for the beverage [23, 26]. These cultures have been piloted for the commercial production of *Obushera* and have promise for use in related fermented food products. However, hitherto this study, no work had been done to establish whether they possess potential probiotic properties. Having been isolated from a traditional fermented food, the starter cultures could be weak or may lack functionality in the human gastrointestinal tract (GIT).

Guidelines for screening candidate microorganisms for probiotic activity have been developed by the Joint FAO/WHO Working Group [27] and Ganguly et al. [28]. Byakika et al. [29] recently reviewed these guidelines. In summary, candidate microorganisms should be screened for the following (1) tolerance to gastrointestinal conditions, (2) safety, and (3) probiotic benefit(s). It was upon these guidelines that the probiotic potentials of *Lb. plantarum* MNC 21, *L. lactis* MNC 24, and *W. confusa* MNC 20 in this study were examined. The reference strain was *Lb. rhamnosus* yoba 2012, which is a known probiotic [30, 31]. The probiotic activity of these starter cultures could translate into technological applications that improve the safety and functionality of fermented foods as well as overall consumer health.

## 2. Materials and Methods

### 2.1. Materials

#### 2.1.1. Microorganisms

*Lb. plantarum* MNC 21, *L. lactis* MNC 24, and *W. confusa* MNC 20 were isolated from *Obushera* [23]. *Lb. rhamnosus* yoba 2012 (originally named *Lb*. *rhamnosus* GG) (Yoba for Life Foundation Amsterdam, The Netherlands) was obtained from the Uganda Industrial Research Institute (IURI), Kampala, Uganda. *E. coli* ATCC 25922, *S. aureus* ATCC 25923, and *S. enterica* were obtained from the College of Veterinary Medicine, Animal Resources and Bio-Security (CoVAB), Makerere University. Stock cultures were stored at −80°C in Ringer's solution containing 15% glycerol. The LAB were independently propagated according to the procedure described by Mukisa [21]. From the stock cultures of *Lb. plantarum* MNC 21, *L. lactis* MNC 24, *W. confusa* MNC 20, and *Lb. rhamnosus* yoba 2012, 0.1 mL was delivered into 100 mL of sterile MRS broth (Laboratorios CONDA, Madrid, Spain) and incubated at 30°C for 24 h. For the *E. coli* ATCC 25922, *S. aureus* ATCC 25923, and *S. enterica,* 0.1 mL of stock cultures was separately inoculated in 100 mL of sterile brain heart infusion (BHI) broth (Laboratorios CONDA, Madrid, Spain) and incubated at 30°C for 24 h. The cells were washed and recovered by centrifugation (7,500x g for 10 min). The cell pellets were suspended in 100 mL of sterile Ringer's solution (Oxoid Ltd, Basingstoke, Hampshire, England) and used for the different screening assays.

### 2.2. Acid and Bile Salt Tolerance

From the fresh culture suspensions, 1 mL of each isolate was separately added to 10 mL of MRS broth (pH = 3.0) acidified using concentrated HCl. The broth was incubated at 30°C for 3 h and cell counts were determined at 0 and 3 h of incubation. Thereafter, 1 mL of the culture from the acidified broth was transferred into 10 mL of MRS broth (pH = 7.8) containing 1% (w/v) ox bile (Oxoid Ltd, Basingstoke, Hants, England). The pH of the broth was adjusted using 1M NaOH. The broth was incubated at 30°C for 9 h. Cell counts were determined at 0, 3, 6, and 9 h of incubation by pour-plating selected serial dilutions in MRS agar (Laboratorios CONDA, Madrid, Spain). Plates were incubated at 30°C for 48 h. Bile salt hydrolase (BSH) activity was also determined according to the method described by Borah et al. [19] with minor modifications. Briefly, freshly grown cultures were spotted on Bile Esculine Agar (Laboratorios CONDA, Madrid, Spain) plates containing 1% ox bile. The plates were incubated at 30°C for 48 h. Hydrolysis of the bile esculine produced a dark brown coloration on the agar. BSH activity was categorized based on the diameter of zones of hydrolysis as: low BSH activity (up to 10 mm), medium BSH activity (11–15 mm), and high BSH activity (>16 mm) [16].

### 2.3. Evaluation of Microbial Safety

#### 2.3.1. Antibiotic Susceptibility

Susceptibility of the isolates to different antibiotics (Bioanalyse, Ankara, Turkey) was determined using the Kirby–Bauer disk diffusion method as previously described [32]. A total of 21 antibiotics (Table 1) were selected from the different classes of antibiotics presented by Charteris et al. [33]. Using sterile cotton swabs, MRS agar plates were swabbed with culture suspensions grown overnight and standardised to 0.5 McFarland (equivalent to 8log cfu/mL). After an hour, antibiotic discs were placed on the surface of the inoculated agar and incubated at 30°C for 48 h. The inhibition zone diameter was measured in mm. The isolates were categorized as resistant, moderately susceptible, or susceptible to the respective antibiotics based on the study by Charteris et al. [33].

#### 2.3.2. Production of Biogenic Amines

Decarboxylation medium was formulated according to Bridson [34]. The medium contained 3 g/L yeast extract (Merck, Darmstadt, Germany), 1 g/L glucose, 0.016 g/L bromocresol purple, and 5 g/L of the corresponding amino acid, all supplied by BDH Laboratory Supplies, Poole, England. The amino acids used were L-histidine, L-tyrosine, L-lysine, L-phenylalanine, L-arginine, and L-ornithine. The pH of the media was adjusted to 6.1 ± 0.2 using 1M NaOH. The medium was subsequently autoclaved at 121°C for 15 min. Ten milliliters of the sterile medium was separately inoculated with 0.1 mL of each isolate (Section 2.1.1), and 1 mL of sterile paraffin was added to the tubes to create anaerobic conditions and avoid false positives. Decarboxylation medium without added amino acids was used as a control. The tubes were incubated at 37°C for 5 days. Decarboxylase activity was indicated by a deep purple coloration.

#### 2.3.3. Mucin Degradation

To examine mucolytic ability of the isolates, the procedure described by Abe et al. [35] was used with some modifications. Mucin obtained from a fresh goat ileum was used. To obtain the mucin, the ileum of a 6-month-old healthy goat was obtained immediately after slaughter from a local abattoir. The ileum was washed in sterile diluent (quarter strength Ringer's solution) to remove ingesta from the mucosal surface and transported to the laboratory in cooled (4°C) sterile diluent. The ileum was cut open, and the mucin was scrapped off using a microscope glass slide. The mucin was centrifuged at 10,000 x g for 10 min to remove epithelial cells and other debris. Thereafter, the supernatant was sterilized by autoclaving at 121°C for 15 min [36]. Mucin sterilized this way does not affect the biological activity of its constituents [37].

To examine the mucolytic ability of the isolates, about 3 log cfu/mL of each culture was separately inoculated in 20 mL of basal media (BM), BM with 1% glucose (BDH Laboratory Supplies, Poole, England), and BM with 0.3% goat mucin. The composition of the BM was 2 g/L yeast extract (Merck, Darmstadt, Germany) and 2 g/L bacteriological peptone. Cultures were incubated at 37°C for 48 h. The cell counts were determined at 0 h and 48 h of incubation. Intestinal microorganisms from a stool sample obtained from a healthy adult volunteer were used as a positive control. The microbes from the stool were first enriched by preculturing in sterile brain heart infusion broth (Laboratorios CONDA, Madrid, Spain) at 37°C for 24 h. For the negative control, a sterile stool sample (heated at 121°C for 20 min) was used. Cell counts were determined by pour-plating selected serial dilutions in sterile MRS agar for the LAB and sterile plate count agar (PCA) (Laboratorios CONDA, Madrid, Spain) for the intestinal microbes. Plates were incubated at 30°C for 48 h. The contribution of substrate (either mucin or glucose) to cell growth was calculated as follows: net growth in basal media with substrate – net growth in basal media.

### 2.4. Evaluation of Potential Probiotic Benefits

#### 2.4.1. Indicators for Ileal Adherence

*(1) Hydrophobicity Assay*. The hydrophobicity of the LAB was evaluated according to methods described by Tomáška et al. [38] and Borah et al. [19] with slight modifications. In brief, washed cultures grown overnight were suspended in sterile quarter strength Ringer's solution. The absorbance (A_0_) of the cell solution at 600 nm was adjusted to 1. From the cell suspension, 6 mL were transferred into a sterile tube and 2 mL of pure xylene (BDH Laboratory Supplies, Poole, England) or pure toluene (Aldrich Chemical Co., Inc, Canada) was added. The mixture was incubated at 25°C for 10 min and then thoroughly vortexed for 2 min. The mixture was left to separate into two phases at 25°C for 20 min. The aqueous phase was carefully removed, and its absorbance (A_1_) was measured at 600 nm. The percentage hydrophobicity was calculated as ((A_0_−A_1_)/A_0_) × 100.

*(2) Auto-Aggregation Assay*. Auto-aggregation was determined according to the procedure described by Kos et al. [39]. From a cell culture grown overnight, 4 mL of the cell suspension (8 log cfu/mL) was vortexed for 10 s and incubated at 25°C for 24 h. At hourly intervals during the 5 h incubation period, 0.1 mL of the upper suspension was added to a tube containing 3.9 mL of quarter strength ringer's solution and its absorbance read at 600 nm. Percentage auto-aggregation was calculated as ((A_0_ – A_1_)/A_0_) × 100, where A_0_ is the absorbance at 0 h and A_1_ is the absorbance at 5 or 24 h.

*(3) Adhesion of LAB to Goat Ileum*. To examine adhesion, the method described by Abbasiliasi et al. [40] was used with some modifications. The goat ileum mentioned in the “mucin degradation” section was used. After it was washed to remove ingesta and delivered to the laboratory, the ileum was cut into several 2.5 cm × 2.5 cm pieces. The pieces were washed by vigorously vortexing thrice in sterile diluent for 10 s to dislodge the inherent microorganisms. To determine the residual microbial counts (C_1_) on the ileum pieces after the third stage of vortexing, selected serial dilutions of the washed ileum were pour plated in sterile PCA. Thereafter, the ileum pieces were separately incubated in a 9 log cfu/mL cell suspension of candidate LAB at 37°C for 45 min. A negative control consisting of the ileum pieces in sterile diluent were also incubated at the same conditions. Thereafter, the pieces were rinsed with sterile diluent to remove the unattached LAB. To determine the counts (C_2_) of the candidate LAB that were attached to the ileum, the pieces were vigorously vortexed in sterile diluent for 10 s to dislodge the microbes. Selected serial dilutions of this diluent were pour plated in sterile PCA, and plates were incubated at 37°C for 48 h. To compute the LAB that were attached onto the ileum, the formula (C_2_ – C_1_)/6.25 cm^2^ was used. The experiment was done in duplicates.

#### 2.4.2. Pathogen Inhibition

*(1) Coaggregation Assay*. Coaggregation was determined following the method described by Kos et al. [39]. From cell cultures grown overnight, 2 mL of each LAB and 2 ml of a specific pathogen (*E. coli* ATCC 25922, *S. aureus* ATCC 25923, or *S. enterica*) were vortexed together for 10 s. The mixture was incubated at 25°C for 5 h. Separate controls containing 4 mL of each individual LAB or the pathogen were also included. The absorbance of the mixed cultures (LAB + pathogen) and single cultures (LAB alone or pathogen alone) were read at 600 nm in a similar manner as in the auto-aggregation assay. The percentage coaggregation was calculated by ((*A*_*x*_ + *A*_*y*_)/2 – A(*x* + *y*)) ÷ ((*A*_*x*_ + *A*_*y*_)/2), where *A*_*x*_ = absorbance of the LAB alone, *A*_*y*_ = absorbance of the pathogenic strain alone, and A(*x* + *y*) = absorbance of the mixture of the LAB and pathogen.

*(2) Production of Antimicrobial Compounds*. Antimicrobial activity was determined using the agar well diffusion assay as described by Vinderola et al. [41] and Jones et al. [42] with modifications. Briefly, LAB were grown overnight in MRS broth (CONDA, Madrid, Spain). The broth was centrifuged at 10,000 x g for 10 min to obtain the cell-free culture supernatant (CFCS). The CFCS was filtered through a 0.45 *µ*m filter (Prat Dumas, France) to remove residual cells. PCA plates were separately swabbed with standardised fresh culture suspensions (adjusted to 10^7^ cfu/mL) of *S. enterica*, *E. coli* ATCC 25922, and *S. aureus* ATCC 25923. A 100 *µ*L of CFCS was placed in 4 mm diameter wells created in the inoculated PCA plates and incubated at 37°C for 24 h. The inhibition zone diameter was measured in mm. To examine if the antimicrobial activity was mediated by organic acids, the pH of the CFCS used was adjusted to 6.5 using 1M NaOH. To test for heat stability of the active antimicrobial compound, CFCS heated at 100°C for 15 min was used. Proteinase K sensitivity was determined using CFCS to which 1 mg/mL of the enzyme (Qiagen Sciences Inc, Germantown, USA) had been added, and the mixture was incubated at 37°C for 3 h.

*(3) Statistical Analyses*. All experiments were performed in duplicate. The data were analyzed using analysis of variance to test for significant differences at an *α* value of 5%. Tukey's HSD test was used to separate the means. Analyses were performed by XLSTAT software (version 2010.5.02, Addinsoft, France).

## 3. Results and Discussion

### 3.1. Acid and Bile Tolerance

The trends in counts of LAB exposed to pH = 3 and 1% bile salts are shown in Table 2. There was a one log decline in cell counts in both treatments throughout the incubation period. Nevertheless, like the reference strain, the counts of starter cultures were above 6 log cfu/mL at the end of the experiments.

Gastric juice and bile salts are biological barriers in the stomach and duodenum, respectively, that can be inhibitory to many microorganisms [43]. Therefore, probiotics need to survive passage through the stomach, where pH can be as low as ≤3.0, and stay alive for 2–4 h [44–46]. Similarly, they must survive passage in the duodenum where bile salt levels can be high as 0.7% [47]. Surviving in these harsh gastric conditions enables probiotics to reach the ileum alive, colonize it, and impart their benefits [48]. Table 2 shows that the starter cultures survived pH = 3 for 3 h and cell counts were still above the minimum (6.0 log cfu/mL) required of probiotic cultures [49]. The survival of microorganisms in gastric juice is attributed to generation of a proton motive force, which expels protons from the cells, thus maintaining a normal intracellular pH [48]. Tolerance to bile is a prerequisite for colonization and metabolic activity of probiotics in the ileum [43, 50]. Bile salts are known to have antimicrobial effects against some microorganisms [51] and may slow down growth [52]. They can disrupt the microbial cellular homeostasis as well as dissociate the lipid bilayer and integrity of the cell membrane resulting in cell death [12]. However, some LAB produce bile salts hydrolase (BSH) which hydrolyzes conjugated bile salts, thus lowering their toxicity [45]. The average bile concentration in the duodenum is about 0.3% [53, 54]. Goldin and Gorbach [55] recommend 0.15–0.3% bile for probiotics screening. In this study, all isolates tolerated broth containing 1% bile salts (Table 2). In fact, at the end of the 9 h of exposure to bile, the counts were above the minimum (6.0 log cfu/mL) required for probiotic effect [49]. These findings are in agreement with those of several authors [54, 56, 57].

#### 3.1.1. Bile Salt Hydrolase (BSH)

The BSH activity of the LAB is summarized in Table 3. The intensity of hydrolysis was in the order as follows: *W. confusa* MNC 20 > *Lb. plantarum* MNC 21 > *L. lactis* MNC 24 > *Lb. rhamnosus* yoba 2012.

BSH is an enzyme produced by intestinal microflora that catalyzes the deconjugation of glycine- or taurine-linked bile salts [58]. The ability of the LAB starters to produce BSH was concomitant with their ability to tolerate bile salts (Table 2). These findings are in agreement with those of other authors who also reported BSH activity in LAB [14, 16, 20, 59]. Deconjugation of bile salts reduces their toxicity, thus enabling probiotic LAB to survive in the duodenum [45, 60]. Deconjugated bile salts are lethal to some pathogens [61], and therefore, BSH activity by the LAB starters could contribute towards their antimicrobial properties. The mechanism by which probiotics protect themselves against these deconjugated salts is yet to be understood. Deconjugated bile salts not only inhibit pathogens but also are associated with reduction of serum cholesterol [4, 61]. This is because deconjugated bile salts are poorly reabsorbed in the liver, which results in their excretion in stool. This increases the demand for serum cholesterol for the *de novo* synthesis of bile salts in the liver [4, 62]. The LAB starters in this study may therefore possess cholesterol-lowering effects. This property is particularly useful for individuals with hypercholesterolemia.

### 3.2. Safety of the LAB Starters

#### 3.2.1. Antibiotic Susceptibility

Table 1 shows the susceptibility of the LAB to different antibiotics. Antibiotic susceptibility categorized as “susceptible,” “moderately susceptible,” and “resistant” was based on the study by Charteris et al. [33]. However, susceptibility to amoxicillin, cephalexin, streptomycin, levofloxacin, and novobiocin against LAB is currently not documented. Therefore, susceptibility to these was based on antibiotics within their classes. Results showed that the *Obushera* LAB starter cultures and reference probiotic were resistant to 10 and 5 of 21 antibiotics, respectively. The *Obushera* starters were generally resistant to penicillin G, ampicillin, vancomycin, gentamycin, kanamycin, tetracycline, ciprofloxacin, metronidazole, sulphamethoxazole-trimethoprin, and colistin. *Lb. rhamnosus* yoba 2012 was only resistant to vancomycin, kanamycin, metronidazole, sulphamethoxazole-trimethoprim, and colistin.

Antibiotic resistance is inherent in some LAB, and the mechanisms involved include absence of a target, low permeability, antibiotic inactivation, and presence of efflux mechanisms, among others [12]. There are concerns that some antibiotic resistant LAB may be reservoirs of antibiotic resistance genes that could be transferred to pathogens [63, 64]. However, with intrinsic resistance, the risk of resistance gene transfer is not only still speculative but also practically impossible [12, 65]. For instance, *Lactobacilli* are known to have a high natural chromosomally encoded resistance to vancomycin [66]. This resistance is due to the absence of *D*-Ala-*D*-lactate in their cell wall which is the target for vancomycin; therefore, such resistance is nontransferable [67, 68]. The natural resistance of *Lactobacilli* to vancomycin could thus explain the results in Table 1. In fact, these results are in agreement with those of Zhou et al. [69] and Leite et al. [70]. In addition, the resistance of *Lactobacilli* to aminoglycosides such as gentamycin, kanamycin, and streptomycin is also thought to be intrinsic by Hummel et al. [71]. The resistance of LAB to penicillin G, ampicillin, vancomycin, gentamycin, kanamycin, tetracycline, ciprofloxacin, metronidazole, sulphamethoxazole-trimethoprin, and colistin is also reported elsewhere [66] [16, 57, 68, 72]. The susceptibility of the LAB species evaluated in this study to some antibiotics such as erythromycin, rifampicin, and chloramphenicol has also been reported in other studies [16, 33, 57, 70].

Antibiotic resistance among probiotic microorganisms could be a desirable trait because it guarantees their survival and thus maintains the natural balance of intestinal microflora even when a host is on antibiotic therapy [73]. This study showed that the starter cultures were resistant to some of the commonly prescribed antibiotics (penicillin G, ampicillin, gentamicin, tetracycline, ciprofloxacin, metronidazole, sulphamethoxazole-trimethoprim, and nitrofurantoin) in Uganda. This implies that administration of these antibiotics in combination with the LAB cultures will not affect their survival in the GIT. However, amoxicillin-clavulanic acid, ceftriaxone, erythromycin, chloramphenicol, rifampicin, or nitrofurantoin treatment will necessitate readministration of the LAB after the antibiotic therapy to allow for recolonization of the intestinal lumen.

#### 3.2.2. Production of Biogenic Amines

The LAB did not decarboxylate L-histidine, L-tyrosine, L-phenylalanine, L-lysine, and L-ornithine. Only *Lb*. *plantarum* MNC 21 and *W*. *confusa* MNC 20 decarboxylated L-arginine, but the extent, indicated by a light purple coloration of the decarboxylation medium, was low.

In order to survive in an acidic environment as is the case in many fermented foods, some LAB decarboxylate amino acids leading to an increase in pH [74]. Additionally, decarboxylation of amino acids results in the formation of biogenic amines: histamine from L-histidine, tyramine from L-tyrosine, cadaverine from L-lysine and L-tyrosine, phenylethylamine from L-phenylalanine and L-ornithine, agmatine from L-arginine, and putrescine from L-phenylalanine and L-ornithine [75]. According to Galgano et al. [76], biogenic amines are produced in all living organisms and play a vital role in cell growth and development. In fact, these amines are also present in the diet but concern is with the amount consumed. Ingestion of biogenic amines in large amounts can trigger toxic reactions such as vasoactivity and psychoactivity affecting the vascular and nervous systems, respectively, [75]. In addition, in the presence of nitrites, biogenic amines can be converted to nitrosoamines in foods [77]. Histamine, tyramine, and phenylethylamine are the biggest concerns, given their serious toxicological effects [75]. On the other hand, putrescine and cadaverine are not toxic but rather enhance the toxic effects of histamine and tyramine [78]. Unfortunately, the threshold of biogenic amine intoxication is difficult to establish since it is dependent on individual responses [79].

Fortunately, the LAB in this study did not produce histamine, tyramine, phenylethylamine, putrescine, or cadaverine. Although production of these biogenic amines was not observed in this study, some researchers have reported otherwise for certain *Lactobacilli* and *Lactococci* strains [79–81]. This illustrates the strain specific nature of this property among microorganisms.

In contrast, *Lb*. *plantarum* MNC 21 and *W*. *confusa* MNC 20 produced agmatine. This is not worrisome because according to Galgano et al. [76], agmatine acts as a neurotransmitter or neuromodulator, stimulates insulin release, and suppresses tumors in the body. In fact, since a limited amount of agmatine is produced in mammalian cells, Galgano et al. [76] suggest that food should contain sufficient amounts to meet the body requirements. Therefore, the ability of *Lb*. *plantarum* MNC 21 and *W*. *confusa* MNC 20 to produce agmatine is a useful trait that could be exploited to enhance agmatine levels in fermented foods.

#### 3.2.3. Mucin Degradation

**Figure 1:**
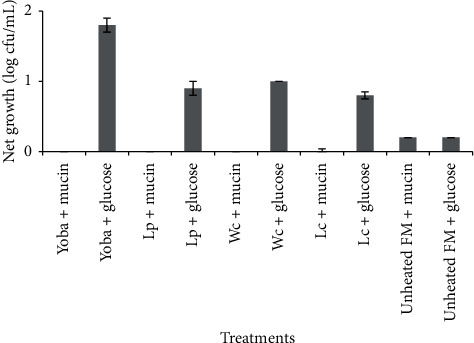
Effect of mucin and glucose on cell growth. Error bars show standard deviations of three independent determinations. Yoba: *Lactobacillus rhamnosus* yoba 2012; Lp: *Lactobacillus plantarum* MNC 21; Wc: *Weissella confusa* MNC 20; *Lc*. *Lactococcus lactis* MNC 24; FM; fecal microbes.

The contribution of mucin and glucose to the growth of LAB is summarized in Figure 1. Results showed that the LAB lacked mucolytic ability. This was shown by the net cell growth in the basal media (BM) and the BM with mucin not being different (*p* > 0.05). Results showed the ability of the unheated fecal microorganims (FM) to grow in the BM containing mucin, an indication of their ability to degrade and utilize mucin as the sole carbon source.

**Figure 2:**
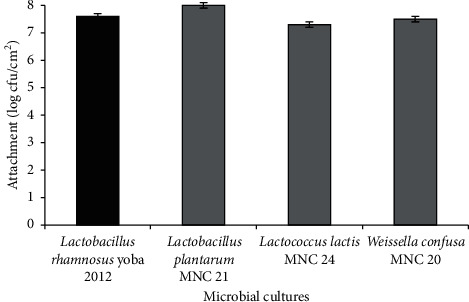
Attachment of lactic acid bacteria to goat ileum. Error bars show standard deviations of three independent determinations.

The GIT is covered by a mucus layer which provides a protective barrier for the underlying epithelium against pathogens and chemical, physical and enzymatic damage [82]. Therefore, microbial mucin degradation is considered a pathogenicity factor since it exposes the intestinal lumen to pathogens [83]. Mucin is an energy source for some intestinal microorganisms, and about 1% of the colonic microbiota can degrade host mucin [84]. The presence of mucin degrading bacteria in the intestine explains the differences in results of the positive and negative controls in this study (Figure 1). Mucolytic ability by mixed intestinal populations has been widely studied [82, 85]. Some specific intestinal microbes with this ability include *Bacteroides fragilis* and *Clostridium perfringens* as well as certain strains of *Bifidobacterium longum, Bifidobacterium bifidum*, and *Bifidobacterium breve* [86–88]. Therefore, given that LAB also form part of the intestinal microbiota, it is important that those intended for probiotic use are screened for mucolytic ability and more so, since the capabilities of all probiotics are not the same [35, 89]. Fortunately, the LAB were unable to degrade mucin (Figure 2). These findings are in agreement with those of other authors [83, 90].

### 3.3. Potential Probiotic Benefits of the LAB Starters

#### 3.3.1. Indicators of Ileal Adherence

*(1) Hydrophobicity and Auto-Aggregation*. The hydrophobicity and auto-aggregation of the LAB are shown in Table 4. *Lb. rhamnosus* yoba 2012 showed the highest (*p* < 0.05) auto-aggregation and hydrophobicity. For the starter cultures, hydrophobicity was in the order of *Lb*. *plantarum* MNC 21 > *W. confusa* MNC 20 > *L. lactis* MNC 24. The hydrophobicity of the starter cultures in either solvent was similar (*p* < 0.05). The highest (*p* < 0.05) auto-aggregation after 5 h of incubation was in *Lb. plantarum* MNC 21. After 24 h of incubation, all isolates had 100% auto-aggregation.

Hydrophobicity is a measure of the relative tendency of a substance to prefer a nonaqueous environment rather than an aqueous environment [91]. Auto-aggregation refers to the aggregation of genetically identical bacteria cells [92]. Both hydrophobicity and auto-aggregation relate to the ability of the microbial cells to adhere to epithelial cells and mucosal surfaces [93, 94]. This is important for formation of biofilms, competition, and colonization in the GIT [95, 96]. It is the hydrophobic nature of the microbial cell surface that enables it attach to the intestinal surface [97]. In this study, the isolates exhibited varying hydrophobicity and auto-aggregation properties, indicating ability to adhere onto the intestinal epithelium and resist the peristaltic movement of food. *Lb. rhamnosus* yoba 2012 was more (*p* < 0.05) hydrophobic than the starter cultures. Nonetheless, the hydrophobicity of the starters in xylene and toluene were similar to values (6–79%) reported for other LAB [39, 62, 98, 99]. Generally, the hydrophobicity was higher (*p* < 0.05) in toluene than in xylene. Indeed, variations in % hydrophobicity of microorganisms in different solvents have been reported [98, 100]. The lack of hydrophobicity among LAB is also documented. Tomáška et al. [38] reported <1% hydrophobicity in some *Lactobacilli*, implying their lack of adhesion properties. The differences in hydrophobicity among LAB are attributed to the extent of expression of the cell surface proteins [101–103].

The hydrophobicity (4.3–18.3%) of the starter cultures were lower than the minimum (40%) that some authors considered while screening for probiotic microorganisms [104]. Indeed, Del Re et al. [105] and Pérez et al. [106] stated that 40% was the minimum for adhesion capabilities. However, the justification for this criterion is not stated. Even so, 4.3–18.3% could be significant in aiding adhesion, and more so, if high doses of the culture are ingested.

Table 4 shows that the starter cultures are strongly self-aggregative moreover, to the same extent as the reference strain. In fact, the results were generally higher than those observed (0–70%) by others [39, 96, 98, 107]. Studies have reported a direct relationship between hydrophobicity and auto-aggregation [98, 104, 108]. However, in this study, hydrophobicity was generally much lower than auto-aggregation. The disparity between hydrophobicity and auto-aggregation confirmed previous findings that hydrophobicity results alone do not necessarily correlate with adhesion properties [97, 107]. Hydrophobicity and auto-aggregation are merely preliminary screening tests for adherent bacteria [105]. Therefore, *ex vivo* or *in vivo* adhesion assays have to be performed to substantiate the adhesion ability.

*(2) Attachment to Goat Ileum*. Figure 2 summarizes the level of attachment of the LAB to the goat ileum. Results indicated the ability of candidate LAB to attach onto the ileum almost to the same extent as the reference strain. *Lb. plantarum* MNC 21 and *L. lactis* MNC 24 showed higher (*p* < 0.05) and similar (*p* > 0.05) attachment than the reference strain, respectively.

Probiotics need to first attach themselves onto the epithelium of the ileum before they can confer health benefits to their host [109]. The attachment to a certain extent enables them to resist the peristaltic movement of the GIT [38]. Additionally, their adhesion displaces pathogens from the luminal-mucosa interface through competition for binding sites on the epithelial and/or mucosal surfaces [94, 110]. Studies have also reported that the adhesion of some probiotic microorganisms onto the ileal epithelium triggers certain immunological responses. Probiotic attachment can also enhance gut barrier function and reduce its permeability to pathogens and antigens [111, 112]. In addition, their attachment to the ileum can stimulate the secretion of antimicrobial compounds by the intestinal epithelial cells [110, 113, 114].

According to Cunha et al. [18], the beneficial effect of a probiotic to its host is related to its concentration in the ileal lumen. The concentration required to obtain a clinical effect is at least 6–7 log cfu/mL or g in the ileum [18, 115]. The ability of the starter cultures to attach onto the epithelium of the ileum at 7.3–8.0 log cfu/cm^2^ (Figure 2) is an excellent property. Even though, according to Pérez et al. [106] and Iñiguez-Palomares et al. [104], the hydrophobicity results of our LAB starters (Table 4) are too low to consider them for adhesion capabilities, results from the *ex vivo* assay (Figure 2) indicate otherwise. This further illustrates that hydrophobicity may not be a good proxy indicator for adhesion to the epithelium.

#### 3.3.2. Pathogen Inhibition

*(1) Coaggregation*. There were variations in the coaggregation abilities of the LAB with *S. aureus* ATCC 25923, *E. coli* ATCC 25922, and *S*. *enterica* (Table 5). *Lb. rhamnosus* yoba 2012 showed the highest (*p* < 0.05) coaggregation with the three isolates. Among the isolates, the highest coaggregations were *W. confusa* MNC 20 with *S. aureus* ATCC 25923 (69.1%), *Lb*. *plantarum* MNC 21 with *S*. *enterica* (61.7%), and *L. lactis* MNC 24 with *E. coli* ATCC 25922 (16.2%). Generally, the isolates coaggregated most with *S*. *enterica* and least with *E*. *coli* ATCC 25922.

Coaggregation is the aggregation of genetically distinct bacterial cells [92]. It is important in preventing the attachment of pathogens onto the intestinal mucosa [116]. Furthermore, through coaggregation, LAB secrete antimicrobial compounds in close proximity with pathogens inhibiting their survival in the GIT [104, 117, 118]. From the results, it appears that *L. lactis* MNC 24, *W. confusa* MNC 20, and *L. plantarum* MNC 21 would be most effective in inhibiting *E. coli*, *Staphylococcus*, and *Salmonella*, respectively. The observed coaggregation of the cultures with *E. coli*, *Staphylococcus*, and *Salmonella* is in agreement with that of other researchers. For instance, coaggregations of our LAB with *S. enterica* and *S*. *aureus* ATCC 25923 (13.8–87.0%) were similar to those (21–49%) of Arief et al. [119]. However, % coaggregations with *E. coli* 25922 (11.3–17.2%) were slightly lower than the values (21–39%) reported by the same authors but were in agreement (15.1%) with those by Kos et al. [39]. The % coaggregation with *S. enterica* (23.8–73.3%) was higher than the value (15.7%) reported by Kos et al. [39]. These discrepancies imply variations in cell surfaces and cell-binding properties of LAB [120].

*(2) Production of Antimicrobial Compounds*. The antimicrobial effect of the LAB is summarized in Table 6. The isolates had an inhibitory effect against *E*. *coli* ATCC 25922, *Staphylococcus aureus* ATCC 25923, and *Salmonella enterica*. Neutralization of the CFCS eliminated its inhibitory effect. Additionally, the action of bacteriocins in the CFCS was not detected.

The antimicrobial action of LAB is attributed to their metabolites, including organic acids, bacteriocins, hydrogen peroxide, ethanol, and diacetyl, among others [25]. The starter culture isolates in this study are fast and high producers of lactic acid [21, 26]. Therefore, lactic acid was possibly the principle microbial inhibitor in this study. In fact, when the pH of CFCS was adjusted to 6.5, its antimicrobial effect disappeared (Table 6). Furthermore, the inability of the heat-and protease-treated CFCS to inhibit microbial growth ruled out the action of bacteriocins. Indeed, similar studies reported that a pH-dependent mechanism was responsible for the antimicrobial activity of LAB [121, 122]. Lactic acid inhibits microorganisms by lowering the extracellular pH and permeabilizing the outer cellular membrane [123]. This collapses the electrochemical proton gradient of the cells which in turn disrupts cellular function and enables the adverse effects of other antimicrobial compounds [123, 124]. The antimicrobial activity of the starter cultures suggests their potential application in promoting the safety of fermented food products.

Although *in vitro* tests are not conclusive means for screening microbes for probiotic properties, they provide an important initial lead. Our findings indicate potential probiotic activity of the *Obushera* starter cultures, but further *in vivo* confirmatory studies are required. The probiotic potential of the *Obushera* LAB only extends to the comparison with a single reference probiotic *Lb. rhamnosus* yoba 2012. Nonetheless, ability of the cultures to tolerate simulated gastrointestinal conditions, pose no safety risk, deconjugate bile salts, adhere to the ileum, and inhibit different pathogenic species is significant in the improvement of health and the safety of fermented food products.

## Figures and Tables

**Table 1:** Susceptibility of lactic acid bacteria to antibiotics.

Antibiotic	Isolate/antibiotic susceptibility			
	*Lactobacillus rhamnosus* yoba 2012	*Weissella confusa* MNC 20	*Lactobacillus plantarum* MNC 21	*Lactococcus lactis* MNC 24
Penicillin G, 10 *µ*g	S	R	R	R
Ampicillin, 10 *µ*g	S	R	R	R
Amoxicillin, 25 *µ*g^*∗∗*^	S	S	S	S
Amoxicillin clavulanic acid, 30 *µ*g	S	S	S	S
Cephalexin, 30 *µ*g	MS^*∗*^	S^*∗*^	S^*∗*^	MS^*∗*^
Vancomycin, 30 *µ*g	R	R	R	R
Ceftriaxone, 30 *µ*g	S	S	S	S
Gentamicin, 10 *µ*g	S	R	R	R
Kanamycin, 30 *µ*g	R	R	R	R
Streptomycin, 300 *µ*g^*∗∗*^	S^*∗*^	S^*∗*^	S^*∗*^	S^*∗*^
Erythromycin, 15 *µ*g	S	S	S	S
Tetracycline, 30 *µ*g	S	R	R	R
Chloramphenicol, 30 *µ*g	S	S	S	S
Ciprofloxacin, 5 *µ*g	S	R	R	R
Levofloxacin, 15 *µ*g	S^*∗*^	S^*∗*^	S^*∗*^	S^*∗*^
Metronidazole, 10 *µ*g^*∗∗*^	R	R	R	R
Sulphamethoxazole-trimethoprim, 25 *µ*g	R	R	R	R
Rifampicin, 5 *µ*g	S	S	S	S
Novobiocin, 30 *µ*g	S^*∗*^	S^*∗*^	S^*∗*^	S^*∗*^
Colistin, 10 *µ*g	R	R	R	R
Nitrofurantoin, 300 *µ*g	S	S	S	S

R: resistant; MS: moderately susceptible; S: susceptible. ^*∗*^Interpretation based on other antibiotics within the same class. ^*∗∗*^Antibiotic concentration used was different from the value indicated by Charteris et al. [33]. Standard antibiotic concentrations were used based on Clinical and Laboratory Standards Institute [32].

**Table 2:** Microbial counts of lactic acid bacteria starters exposed to high acid (pH = 3) and 1% bile.

Time (h)	Microbial counts (log cfu/mL)							
	*Lactobacillus rhamnosus* yoba 2012		*Weissella confusa* MNC 20		*Lactobacillus plantarum* MNC 21		*Lactococcus lactis* MNC 24	
	pH = 3	1% bile	pH = 3	1% bile	pH = 3	1% bile	pH = 3	1% bile
0	8.7^a^ ± 0.0	8.2^a^ ± 0.1	9.0^a^ ± 0.0	8.1^a^ ± 0.0	8.9^a^ ± 0.0	8.8^a^ ± 0.1	8.5^a^ ± 0.1	8.6^a^ ± 0.0
3	7.7^b^ ± 0.1	8.1^ab^ ± 0.0	8.4^b^ ± 0.0	8.0^a^ ± 0.0	8.1^b^ ± 0.1	8.3^b^ ± 0.1	7.2^b^ ± 0.1	8.4^b^ ± 0.0
6		7.6^b^ ± 0.0		7.6^b^ ± 0.0		7.8^c^ ± 0.0		8.0^c^ ± 0.0
9		7.2^c^ ± 0.0		7.5^b^ ± 0.0		7.3^d^ ± 0.0		7.8^d^ ± 0.0

Values are means ± standard deviations of three independent determinations. Mean values in the same column with the same superscripts are not significantly different (*p* > 0.05).

**Table 3:** Bile salt hydrolase activity of lactic acid bacteria isolates.

Lactic acid bacteria	Hydrolysis zone (mm)	BSH activity
*Lb. rhamnosus* yoba 2012	6.3^d^ ± 1.1	Low
*L. lactis* MNC 24	7.3^c^ ± 1.4	Low
*Lb. plantarum* MNC 21	10.7^b^ ± 1.4	Medium
*W. confusa* MNC 20	12.6^a^ ± 1.7	Medium

Values are means ± standard deviations of three independent determinations. Values in the same column with the same superscripts are not significantly different (*p* > 0.05). BSH activity was categorized based on diameter of zones of hydrolysis as low BSH activity (up to 10 mm), medium BSH activity (11–15 mm), and high BSH activity (>16 mm) [16].

**Table 4:** Auto-aggregation and hydrophobicity of lactic acid bacteria.

Lactic acid bacteria	% hydrophobicity		% auto-aggregation	
	Xylene	Toluene	5 h	24 h
*Lactobacillus rhamnosus* yoba 2012	80.3^a^ ± 3.2	66.3^a^ ± 2.8	100.0^a^ ± 0.0	100.0^a^ ± 0.0
*Lactobacillus plantarum* MNC 21	15.9^b^ ± 1.5	12.6^b^ ± 1.7	100.0^a^ ± 0.0	100.0^a^ ± 0.0
*Lactococcus lactis* MNC 24	5.5^c^ ± 2.7	5.9^d^ ± 2.4	75.8^c^ ± 2.9	100.0^a^ ± 0.0
*Weissella confusa* MNC 20	4.3^d^ ± 0.5	8.4^c^ ± 2.7	79.9^d^ ± 2.0	100.0^a^ ± 0.0

Values are means ± standard deviations of three independent determinations. Mean values in the same column with similar superscripts are not significantly different (*p* > 0.05).

**Table 5:** Percentage coaggregation of lactic acid bacteria with selected pathogens.

Lactic acid bacteria	Pathogenic bacteria		
	*Escherichia coli* ATCC 25922	*Staphylococcus aureus* ATCC 25923	*Salmonella enterica*
*Lb. rhamnosus* yoba 2012	17.2^a^ ± 0.2	87.0^a^ ± 0.2	73.3^a^ ± 0.2
*Lb. plantarum* MNC 21	12.6^c^ ± 0.1	33.8^c^ ± 0.2	61.7^b^ ± 0.1
*W. confusa* MNC 20	11.3^d^ ± 0.0	69.1^b^ ± 0.0	56.4^c^ ± 0.3
*L. lactis* MNC 24	16.2^b^ ± 0.2	13.8^d^ ± 0.1	23.8^d^ ± 0.1

Values are means ± standard deviations of three independent determinations. Mean values in the same column with same superscripts are not significantly different (*p* > 0.05).

**Table 6:** Antimicrobial activity of lactic acid bacteria isolates.

Cell-free culture supernatant treatment	*Escherichia coli* ATCC 25922	*Staphylococcus aureus* ATCC 25923	*Salmonella enterica*
No treatment			
*Lactobacillus rhamnosus* yoba 2012	+	+	+
*Weissella confusa* MNC 20	+	+	+
*Lactobacillus plantarum* MNC 21	+	+	+
*Lactococcus lactis* MNC 24	+	+	+

pH = 6.5			
*Lactobacillus rhamnosus* yoba 2012	−	−	−
*Weissella confusa* MNC 20	−	−	−
*Lactobacillus plantarum* MNC 21	−	−	−
*Lactococcus lactis* MNC 24	−	−	−

100°C for 15 min			
*Lactobacillus rhamnosus* yoba 2012	+	+	+
*Weissella confusa* MNC 20	+	+	+
*Lactobacillus plantarum* MNC 21	+	+	+
*Lactococcus lactis* MNC 24	+	+	+

Proteinase K (37°C, 3 h)			
*Lactobacillus rhamnosus* yoba 2012	+	+	+
*Weissella confusa* MNC 20	+	+	+
*Lactobacillus plantarum* MNC 21	+	+	+
*Lactococcus lactis* MNC 24	+	+	+

Inhibition zone diameter: –(<11 mm); +(11–16 mm) [16].

## Data Availability

The data in tables and figures used to support the findings of this study are included within the article.
